# Low Dielectric Medium for Hyperbolic Phonon Polariton Waveguide in van der Waals Heterostructures

**DOI:** 10.3390/nano14161344

**Published:** 2024-08-14

**Authors:** Byung-Il Noh, Salvio Reza, Cassie Hardy, Jiahan Li, Adib Taba, Masoud Mahjouri-Samani, James H. Edgar, Siyuan Dai

**Affiliations:** 1 Materials Research and Education Center, Department of Mechanical Engineering, Auburn University, Auburn, AL 36849, USA; cmh0205@auburn.edu; 2 Department of Physics, Auburn University, Auburn, AL 36849, USA; ssr0024@auburn.edu; 3 Tim Taylor Department of Chemical Engineering, Kansas State University, Manhattan, KS 66506, USA; jiahanli@ksu.edu (J.L.); edgarjh@ksu.edu (J.H.E.); 4 Department of Electrical and Computer Engineering, Auburn University, Auburn, AL 36849, USA; taba.adib@auburn.edu (A.T.); mzm0185@auburn.edu (M.M.-S.)

**Keywords:** s-SNOM, hyperbolic phonon polaritons, hexagonal boron nitride, van der Waals materials, optical confinement, waveguide

## Abstract

Polar van der Waals (vdW) crystals, composed of atomic layers held together by vdW forces, can host phonon polaritons—quasiparticles arising from the interaction between photons in free-space light and lattice vibrations in polar materials. These crystals offer advantages such as easy fabrication, low Ohmic loss, and optical confinement. Recently, hexagonal boron nitride (hBN), known for having hyperbolicity in the mid-infrared range, has been used to explore multiple modes with high optical confinement. This opens possibilities for practical polaritonic nanodevices with subdiffractional resolution. However, polariton waves still face exposure to the surrounding environment, leading to significant energy losses. In this work, we propose a simple approach to inducing a hyperbolic phonon polariton (HPhP) waveguide in hBN by incorporating a low dielectric medium, ZrS_2_. The low dielectric medium serves a dual purpose—it acts as a pathway for polariton propagation, while inducing high optical confinement. We establish the criteria for the HPhP waveguide in vdW heterostructures with various thicknesses of ZrS_2_ through scattering-type scanning near-field optical microscopy (s-SNOM) and by conducting numerical electromagnetic simulations. Our work presents a feasible and straightforward method for developing practical nanophotonic devices with low optical loss and high confinement, with potential applications such as energy transfer, nano-optical integrated circuits, light trapping, etc.

## 1. Introduction

Phonon polaritons, quasiparticles arising from the interaction between free-space photons and lattice vibrations in polar materials such as alpha-phase molybdenum trioxide (α-MoO_3_), hexagonal boron nitride (hBN), silicon carbide (SiC), perovskite oxide compound (ABO_3_), etc. [1,2,3,4,5,6,7], exhibit a remarkable ability to confine incident electromagnetic (EM) waves. The polariton wavelength (*λ*_p_) is considerably smaller than the free-space light wavelength (*λ*_0_), facilitating the control of light-matter interactions at the nanoscale. This advance has promoted cutting-edge optics research, including topological photonics, light emission, the manipulation of nano-light, and so on [8,9,10,11,12,13].

Recently, hBN, one of the natural van der Waals (vdW) materials with anisotropic lattice vibrations, has garnered attention due to its advantageous properties, including immunity to Ohmic loss, substantial optical confinement, easy fabrication, and more. Notably, hBN exhibits a unique characteristic known as hyperbolicity [14]. This is manifested in its permittivity tensors along orthogonal axes displaying opposite signs (ε_i_ε_z_ < 0, *i* = *x*, or *y*) within specific frequency ranges, namely Reststrahlen (RS) band 1 (780–830 cm^−1^, where ε_i_ > 0 and ε_z_ < 0) and RS band 2 (1370–1610 cm^−1^, where ε_i_ < 0 and ε_z_ > 0). The inherent hyperbolicity in hBN gives rise to multiple branches of hyperbolic phonon polaritons (HPhPs). These branches not only result in exceptionally high energy density but also enable optical engagement at the atomic scale, facilitating significant optical confinement. These merits have provided the possibility of utilizing advanced nanophotonic applications.

To implement practical polaritonics, a specific design is essential to mitigate undesired energy loss during HPhP propagation. Some studies have introduced guided HPhPs in hBN through designs like patterned ribbons [15,16] and in-plane refractive mediums [17,18]. However, the former approach typically required the etching of polaritonic materials, resulting in significant losses due to material damage. Additionally, the latter approach utilized phase-change materials (VO_2_) to manipulate HPhP guidance, but this could also induce optical loss in the specific phase. Furthermore, HPhPs can be vulnerable to challenging environments including features such as high temperature, humidity, and exposure to certain chemicals, which can cause optical losses. Therefore, minimizing external influences is important for developing practical polaritonic devices.

Here, we propose vdW heterostructures that incorporate a low dielectric medium between hBN slabs, forming HPhP waveguides. Zirconium disulfide (ZrS_2_), a two-dimensional transition metal dichalcogenide (TMDC) with low permittivity in the upper RS band 2 of hBN, is regarded as a promising candidate for the waveguide medium. This can effectively mitigate optical losses induced by the external environment while concurrently serving as a guiding medium for highly confined HPhPs. The experimental and theoretical confirmation of ZrS_2_ as the low dielectric medium for guided HPhPs is achieved by varying thicknesses of ZrS_2_. This validation is conducted using advanced instruments such as Scattering-type Scanning Near-Field Optical Microscopy (s-SNOM) and numerical EM simulations (COMSOL Multiphysics). Note that ZrS_2_ not only supports condensed HPhP waveguides to prevent undesired interaction with the surrounding medium but also facilitates highly confined EM fields. The experimental and simulation data, extracted through Fourier Transform (FT) analysis of line profiles from s-SNOM and COMSOL simulation, align impeccably with EM calculations of the complex reflectivity Im *r*_p_ of the hBN/ZrS_2_/hBN heterostructure in the energy (*ω*)–momentum (*k*_p_/*k*_0_) dispersion. Our vdW heterostructure presents a straightforward design for nano-polaritonic devices, offering low loss and high field confinement of free-space light. This design holds promise for applications in nano-optical integrated circuits, energy transfer optoelectronics, and various other fields.

## 2. Materials and Methods

### 2.1. Sample Preparation

The hBN bulk crystals were grown at atmospheric pressure by precipitation from molten metal solutions [19]. The hBN and ZrS_2_ (2D Semiconductor Inc., Scottsdale, AZ, USA) were mechanically exfoliated from bulk source crystals and transferred to cleaned Si wafers covered with 300 nm thick thermal silicon dioxide (SiO_2_, UniversityWafer Inc., Boston, MA, USA) by using white tapes, respectively. In order to stack each flake, a Poly(bisphenol A carbonate) (PC, analytical standard, Sigma-Aldrich, St. Louis, MO, USA)/Polydimethylsiloxane (PDMS) stamp was prepared. The PC solutions (6 wt%) were obtained by fully dissolving PC powders in chloroform (≥99.5%, Sigma-Aldrich, USA) at room temperature for an hour. Next, several PC droplets were poured into a glass piece and covered with another glass piece. Then, the top glass piece was quickly detached and dried to remove the solvent. The PC/PDMS stamp was finally prepared by covering a PC film over 1 × 1 μm^2^ PDMS and fixing it with tape.

### 2.2. Fabrication of van der Waals Heterostructures

The hBN/hBN, hBN/10 nm ZrS_2_/hBN, and hBN/43 nm ZrS_2_/hBN vdW heterostructures were fabricated simultaneously using the dry-transfer method. A customized transfer stage, connected to an optical microscope, enabled the precise positioning of each flake for hBN/hBN, hBN/10 nm ZrS_2_/hBN, and hBN/43 nm ZrS_2_/hBN configurations. The fabrication process involved picking up the top hBN flake by contacting the PC/PDMS stamp, followed by sequentially picking up ZrS_2_ in the same manner at 50 °C. Subsequently, we attached ZrS_2_/hBN/PC/PDMS to the bottom hBN and heated the hot plate to 140 °C for a couple of minutes. During this process, the PC films detached from PDMS and remained with hBN/ZrS_2_/hBN on the wafer. Finally, hBN/hBN, hBN/10 nm ZrS_2_/hBN, and hBN/43 nm ZrS_2_/hBN configurations were obtained by removing PC films in a chloroform solution at room temperature for 15 min.

### 2.3. Near-Field Infrared Radiation Nano-Imaging

The HPhPs in vdW heterostructures were imaged using a commercial s-SNOM (www.neaspec.com accessed on 10 May 2024) based on a tapping-mode atomic force microscope (AFM). The Pt/Ir-coated silicon AFM tip (tip radius~25 nm) was purchased from NanoAndMore (https://www.nanoandmore.com/AFM-Probe-ARROW-NCPt accessed on 3 March 2024). The AFM tip was illuminated by a monochromatic mid-IR quantum cascade laser (QCL) (www.daylightsolutions.com accessed on 25 January 2024) with frequency ω_0_ spanning from 845 to 1800 cm^−1^ (*p*-polarized). The s-SNOM images were recorded by a pseudoheterodyne interferometric detection module with an AFM tapping frequency of 250–280 kHz and a tapping amplitude of around 70 nm. The s-SNOM output signal was demodulated at the third (3th) harmonic of the tapping frequency to reduce the background signal.

### 2.4. Full-Wave Electromagnetic Numerical Simulation

Full-wave EM numerical simulations for our heterostructures in this study were performed using COMSOL Multiphysics. hBN permittivity (Table 1) was obtained using the Lorentz model (Supplementary Information S1). Ports were implemented in the cross-section of our heterostructure (Table 2) to launch the polaritons from the left side (Port 1) and collect the polaritons at the right side (Port 2). The COMSOL images revealed the wave properties of HPhPs. The field distribution of the absolute value of the electric field (|E_z_|) was extracted perpendicular to the vdW heterostructures.

## 3. Results and Discussion

**Figure 1:**
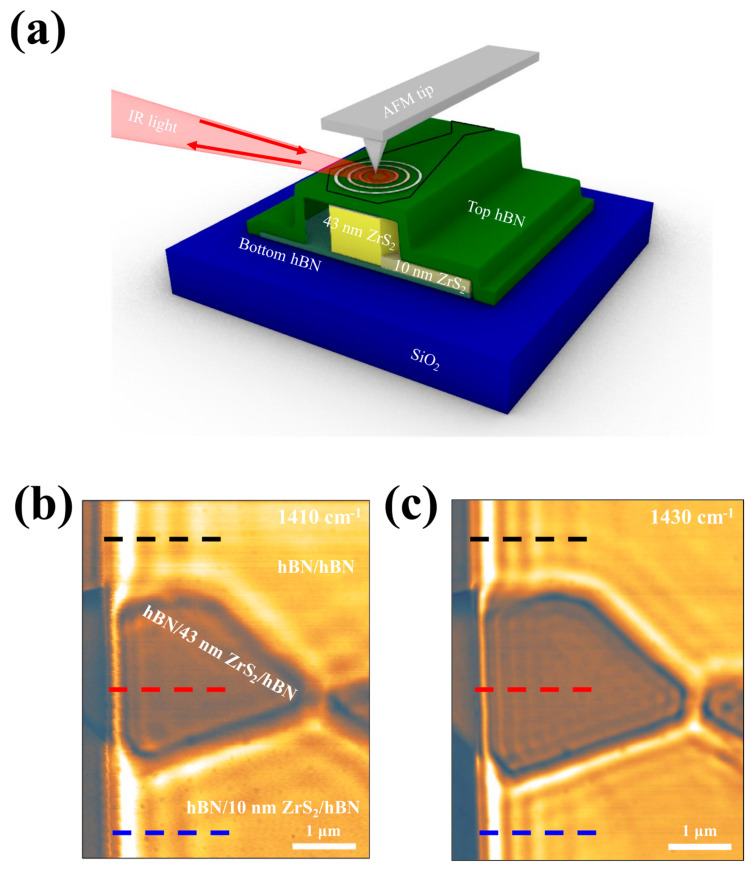
Schematic illustration and nano-imaging of van der Waals (vdW) heterostructures. (**a**) Schematics of the experimental setup to image the hyperbolic phonon polariton (HPhP) waveguide with and without low dielectric medium, ZrS_2_. The AFM tip is illuminated by broad mid-infrared (IR) beams from quantum cascade lasers (QCL), which excites HPhP. The back-scattered IR signals are collected to draw s-SNOM images. Experimental s-SNOM images of the vdW heterostructures at the illuminating frequencies ω = 1410 cm^−1^ (**b**) and ω = 1430 cm^−1^ (**c**). The colorful dashed lines correspond to hBN/hBN (black), hBN/43 nm ZrS_2_/hBN (red), and hBN/10 nm ZrS_2_/hBN (blue) regions, respectively.

The HPhP waveguide within the low dielectric medium was visualized by s-SNOM (Figure 1a). The AFM tip was illuminated by incident free space of IR light *λ*_0_ at RS band 2 frequency ranges ω = 1360–1650 cm^−1^ and a strong optical near-field was created between the AFM tip and the surface of vdW heterostructures, launching the HPhPs in the vdW heterostructures. The heterostructures comprise three regions: hBN/hBN, hBN/10 nm ZrS_2_/hBN, and hBN/43 nm ZrS_2_/hBN. The thicknesses of each vdW block were 11.5, 6.5, 10, and 43 nm, corresponding to top hBN, bottom hBN, thin ZrS_2_, and thick ZrS_2_ (Figure S2). The HPhPs propagated to the edge and were reflected, interfering with propagating HPhPs from the AFM tip. This interference formed a standing wave in the s-SNOM images at a 10 nm spatial resolution. The s-SNOM images of hBN/hBN, hBN/10 nm ZrS_2_/hBN, and hBN/43 nm ZrS_2_/hBN at the frequency ω = 1410 and 1430 cm^−1^ in Figure 1b,c display fringes of HPhPs from each region. The parallel oscillation period, shown as a standing wave in the s-SNOM image, represents the wavelength (*λ*_p_) of the HPhP and exhibits the HPhP starting from the crystal edge with a gradual decay. At ω = 1410 cm^−1^, the wavelength of HPhPs in hBN/10 nm ZrS_2_/hBN was slightly shorter than hBN/hBN. At ω = 1410 cm^−1^, the wavelength of HPhPs in hBN/10 nm ZrS_2_/hBN was slightly shorter than hBN/hBN. Unlike the two aforementioned regions, the wavelength of HPhPs in the hBN/43 nm ZrS_2_/hBN region appeared much shorter and had a lower intensity. At ω = 1430 cm^−1^ (Figure 1c), all three regions vividly exhibited HPhPs in the s-SNOM image and the wavelength of HPhPs was shorter than at ω = 1410 cm^−1^ (Figure 1b). Among them, the wavelength of HPhPs in hBN/10 and 43 nm ZrS_2_/hBN was shorter than hBN/hBN due to the dielectric environment effect. Typically, HPhPs of hBN at a high frequency become highly damped and have a short wavelength due to strong interactions with dielectric materials. However, at a relatively higher frequency, the oscillation period of the fringes in the hBN/43 nm ZrS_2_/hBN region was clearly visualized and their numbers appeared larger than at low frequency. Therefore, we anticipated the emergence of different light guide modes in the hBN/43 nm ZrS_2_/hBN region.

**Figure 2:**
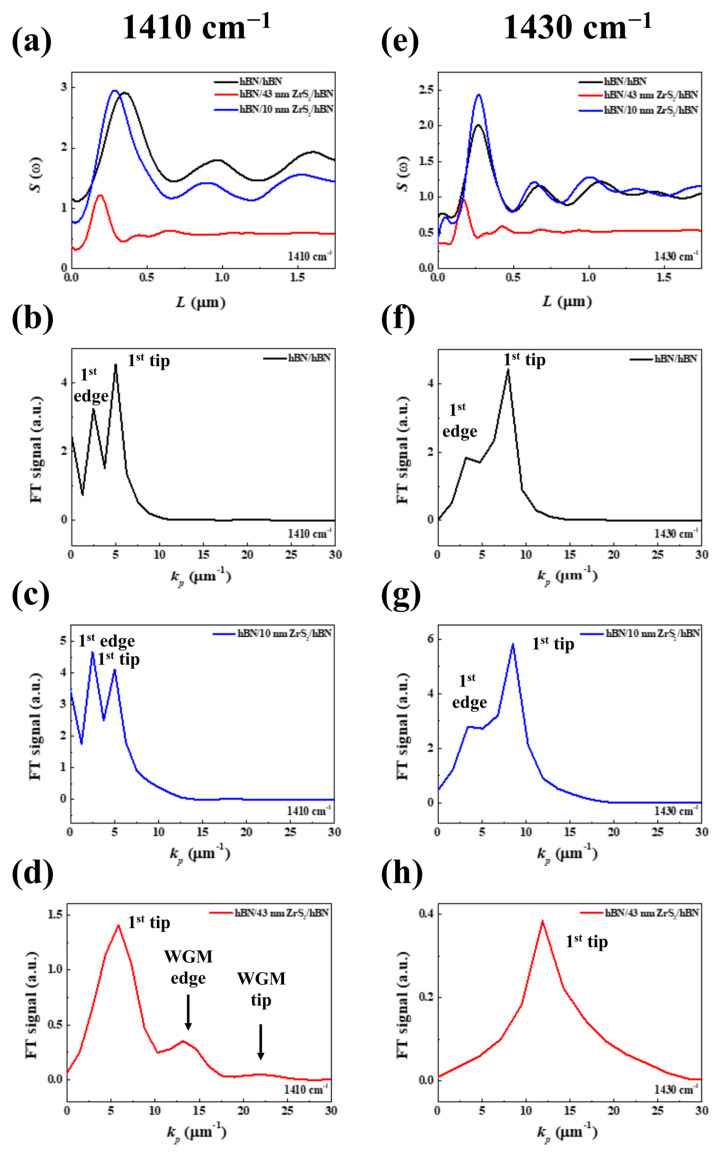
HPhP line traces and Fourier Transform (FT) spectra. (**a**,**e**) s-SNOM profiles cut along the edge from Figure 1b,c in hBN/hBN, hBN/43 nm ZrS_2_/hBN, and hBN/10 nm ZrS_2_/hBN at ω = 1410 cm^−1^ and 1430 cm^−1^. (**b**–**d**,**f**–**h**) FT spectra of the s-SNOM line profile in (**a**,**e**). The two peaks indicate the 1st branch of the edge- and tip-launched hBN mode in the hBN/10 nm ZrS_2_/hBN heterostructure. In the hBN/43 nm ZrS_2_/hBN heterostructure, the FT peaks correspond to the hBN mode and the ZrS_2_ waveguide mode (WGM).

For the quantification of HPhP behaviors, s-SNOM line profiles were extracted by cutting dashed lines from each region in the s-SNOM images (Figure 2). HPhPs were regarded as EM waves, and the surrounding dielectric medium could influence their polariton confinement and propagation length [20]. The wavelengths of hBN/hBN, hBN/10 nm ZrS_2_/hBN, and hBN/43 nm ZrS_2_/hBN at ω = 1410 and 1430 cm^−1^ were 1.266, 1.218, 0.808 μm and 0.831, 0.736, 0.494 μm, respectively. Due to the slightly higher permittivity of ZrS_2_ (ɛ_ZrS2_ ≈ 2.2) [21] compared to air (ɛ_Air_ = 1), hBN/10 nm ZrS_2_/hBN and hBN/43 nm ZrS_2_/hBN exhibited shorter wavelengths than hBN/hBN. Notably, the wavelengths of HPhPs in hBN/43 nm ZrS_2_/hBN at two frequencies were much shorter than in other regions, which may be expected to cause other phenomena in addition to dielectric environment effects (Figure 2a,e). To classify different modes in the three regions at both frequencies, the momentum *k*_p_ (2π/*λ*_p_) corresponding to each HPhP mode was obtained by separating the s-SNOM line profiles via FT analysis. FT curves in Figure 2b,c,f,g revealed edge- and tip-launched HPhPs, typical features in polaritonic materials. In Figure 2d, three FT peaks at *k*_p_ = 6, 12, and 23 µm^−1^ represent the tip-launched hBN mode, edge-launched ZrS_2_ waveguide mode, and tip-launched ZrS_2_ waveguide mode, respectively [22]. In Figure 2h, the FT peak indicates the tip-launched hBN mode in the top and bottom hBN slabs. This was notably different from other regions and frequencies.

**Figure 3:**
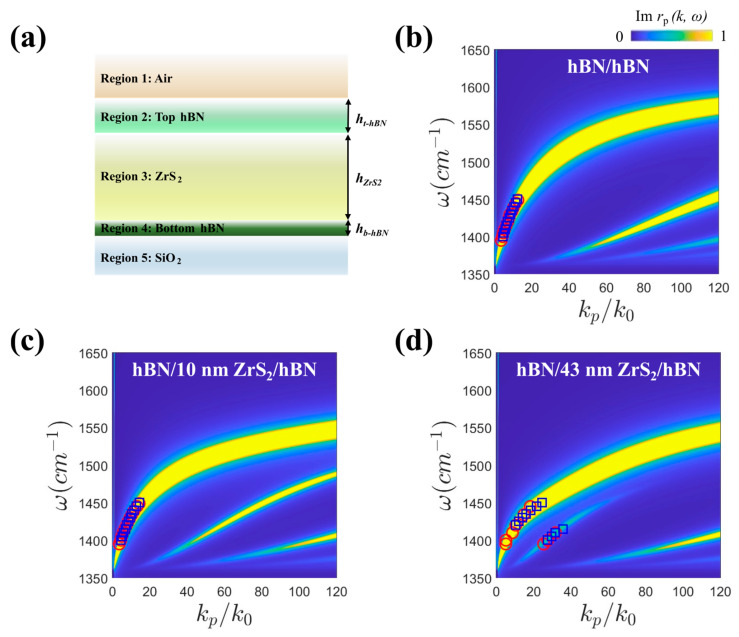
Energy (ω)–momentum (*k*_p_/*k*_0_) dispersions in vdW heterostructures. (**a**) Schematics of a multilayer system of hBN/ZrS_2_/hBN heterostructures. (**b**–**d**) The calculated false-color map is the calculated imaginary reflectivity Im *r*_p_ in hBN/hBN, hBN/10 nm ZrS_2_/hBN, and hBN/43 nm ZrS_2_/hBN. The experimental data (red circle) from s-SNOM images and the numerical simulation (blue square) are plotted in the ω − *k*_p_/*k*_0_ dispersion.

To investigate this unusual phenomenon, a false-color dispersion of ω − *k*_p_/*k*_0_ for each region in the heterostructures is presented, covering frequency ranges of ω = 1360–1650 cm^−1^ (Figure 3) [23,24]. The dispersion comprises multilayer systems (Figure 3a), and the imaginary part of the complex reflectivity Im *r*_p_ in the vdW heterostructures clearly visualizes multiple polariton branches (Supplementary Information S2). The experimental (red circle) and theoretical (blue square) confinement, calculated through s-SNOM imaging and COMSOL simulation, respectively, excellently agreed with Im *r*_p_ in the false-color dispersion of hBN/hBN, hBN/10 nm ZrS_2_/hBN, and hBN/43 nm ZrS_2_/hBN (Figure 3b–d). The wavelength of the EM wave is regarded as the distance between two peaks in the line profiles from the COMSOL images (Figure S3). The theoretical confinement values were calculated from the wavelengths extracted above 15 nm from the top hBN. Compared to Figure 3b,c, in Figure 3d, the false-color dispersions of hBN/43 nm ZrS_2_/hBN differed significantly, despite having the same structural configuration as hBN/10 nm ZrS_2_/hBN. Due to the electromagnetic interaction between the separated top and bottom hBN slabs, the first branch of the HPhPs splits into two modes (Figure 3d): one is confined to the top and bottom hBN (hBN mode), and the other one is guided by ZrS_2_ (ZrS_2_ waveguide mode), represented by the first and second branches in Figure 3d, respectively.

**Figure 4:**
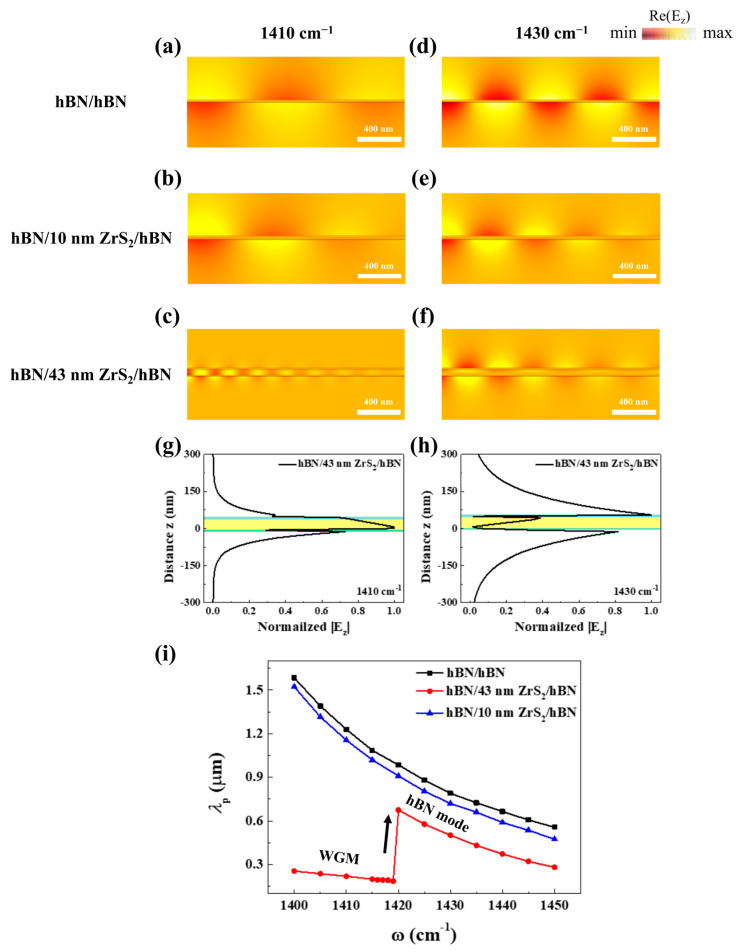
Full-wave electromagnetic (EM) numerical simulation of vdW heterostructures. (**a**–**f**) The cross-sectional view of the real space images of Re(E_z_) fields on hBN/hBN, hBN/10 nm ZrS_2_/hBN, and hBN/43 nm ZrS_2_/hBN at ω = 1410 (**a**–**c**) and 1430 cm^−1^ (**d**–**f**). Theoretical EM field distribution |E_z_| profiles of hBN/43 nm ZrS_2_/hBN at ω = 1410 (**g**) and 1430 cm^−1^ (**h**). Shaded areas mark the top hBN (cyan)/middle 43 nm ZrS_2_ (yellow)/bottom hBN (green), respectively. (**i**) The dependence of the theoretical HPhP wavelength *λ*_p_ on the frequency ω.

To validate the experimental results and our predictions, we conducted full-wave numerical simulations using the commercial software COMSOL Multiphysics 6.0, shown in Figure 4. In Figure 4a–f, the cross-sectional views of the real space images of Re(E_z_) fields for hBN/hBN, hBN/10 nm ZrS_2_/hBN, and hBN/43 nm ZrS_2_/hBN at 1410 and 1430 cm^−1^ are visualized, respectively. In Figure 4a,b,d–f, the simulated EM waves of Re(E_z_) fields appear in the same form as the HPhPs, displaying the same EM field distribution as the single-slab hBN. Additionally, in Figure 4i, the curves of their simulated wavelengths gradually decreased, exhibiting the same tendency as the false-color dispersion and experimental data. In the real-space image of Re(E_z_) and |E_z_| profiles of hBN/43 nm ZrS_2_/hBN at 1410 cm^−1^, the HPhP waveguide with highly confined EM fields inside the thick ZrS_2_ (43 nm) emerged due to the strong coupling between the hBN modes of the top and bottom hBN layers (Figure 4c,g). This HPhP waveguide in the 43 nm ZrS_2_ persisted up to the frequency ω = 1420 cm^−1^ (Figure 4i) and then transitioned to typical HPhPs (Figure 4h and Figure S4). The formation of the HPhP waveguide depended on the permittivity and thickness of the materials, along with the propagation constant [25]. As the permittivity increases (Re(*ε*_MoS2_) = 15 and Re(*ε*_PtSe2_) = 26), it becomes more difficult to form the waveguide (Figure S5) [26,27]. Clearly, the selection of a dielectric medium is a crucial consideration for HPhP waveguides in vdW heterostructures.

## 4. Conclusions

In conclusion, we introduce vdW heterostructures designed for HPhP waveguides within a low dielectric medium ZrS_2_. The HPhP waveguide was achieved through both near-field optical nano-imaging and numerical EM simulation. Moreover, our experimental results on EM energy confinement, dependent on frequency, matched the dispersion relation very well. We further established that the transformation of the polariton guide form at a given frequency is intricately linked to the propagation constant, relying on the permittivity and layer thickness. In our experiments, we characterized the ZrS_2_ HPhP waveguide in the range of 1370–1420 cm^−1^. This waveguide is expected to cover a broader frequency range by optimizing the design of the heterostructure. Our work presents a viable design for nano-polaritonics with a high energy confinement of free-space light. Compared to other phonon polaritonic materials such as α-MoO₃ and SiC, the hBN/ZrS_2_/hBN waveguide shares similar characteristic frequencies with a variety of organic compounds, thereby enabling fingerprinting for material identification and many other important functionalities. This highlights the significance of guide medium selection in providing tunable nanophotonic devices. In addition to encapsulated-type hyperbolic waveguides, strip-type hyperbolic waveguides without the core layer are also valuable for potential applications in nano-optical integrated circuits, energy transfer optoelectronics, and various other advanced fields. Furthermore, it is worth exploring HPhP waveguides with negative dispersion for potential applications in backward couplers [28], cloaking [29], etc. [30,31].

## Figures and Tables

**Table 1:** Complex permittivity of each material.

ω (cm^−1^)	ε_hBN,x_	ε_hBN,z_	ε_SiO2_	ε_ZrS2_
1410	−21.69 + 1.895i	2.322 + 0.0002i	1.07	2.2 + 0.0001i
1430	−14.003 + 0.969i	2.743 + 0.0006i		

**Table 2:** Dimension of each material.

Dimension	Top hBN	ZrS_2_	Bottom hBN	SiO_2_
Width	10 μm			
Thickness	11.5 nm	0/10/43 nm	6.5 nm	300 nm

## Data Availability

The data presented in this study are available on request from the corresponding authors.

## Supplementary Materials

The following supporting information can be downloaded at: https://www.mdpi.com/article/10.3390/nano14161344/s1, Figure S1: Optical information of hBN. (a) Raman spectrum of the natural hBN. (b,c) Real and imaginary parts of the dielectric function for natural hBN. Figure S2: Morphology characterization. (a) AFM image of vdW heterostructures in this work. (b) Height profiles were obtained from several dashed lines in the left panel (a). Figure S3: Line profiles of Re(E_z_) from COMSOL simulation. hBN/hBN, hBN/10 nm ZrS_2_/hBN, and hBN/43 nm ZrS_2_/hBN at ω = 1410 (a–c) and 1430 cm^−1^ (d–f). Figure S4: Theoretical EM field distribution |E_z_| profiles. hBN/hBN and hBN/10 nm ZrS_2_/hBN at ω = 1410 cm^−1^ (a,b) and 1430 cm^−1^ (c,d). Figure S5: Permittivity dependence. Full-wave EM numerical simulation of vdW heterostructures inserting different middle layers: 43 nm of (a) air, (b) ZrS_2_, (c) MoS_2_, and (d) PtSe_2_ at the frequency ω = 1410 cm^−1^. Table S1: Parameters for the calculated complex dielectric function of hBN. Reference [32] is cited in the Supplementary Materials.
