# Supplementary material for: Low Dielectric Medium for Hyperbolic Phonon Polariton Waveguide in van der Waals Heterostructures
Source: Nanomaterials (Basel). 2024 Aug 14;14(16):1344. doi: 10.3390/nano14161344 (PMC11356932; doi:10.3390/nano14161344)
Supplement: Supplementary file 1 [file nanomaterials-14-01344-s001.zip › nanomaterials-3091940-supplementary.pdf]

Low Dielectric Medium for Hyperbolic Phonon Polariton Waveguide in van der Waals Heterostructures

Byung-Il Noh [1,*], Salvio Reza [2], Cassie Hardy [1], Jiahan Li [3], Adib Taba [4], Masoud Mahjouri-Samani [4], James H Edgar [3] and Siyuan Dai [1,*]

[1] Materials Research and Education Center, Department of Mechanical Engineering, Auburn University, Auburn, AL 36849, USA; cmh0205@auburn.edu
[2] Department of Physics, Auburn University, Auburn, AL 36849, USA; ssr0024@auburn.edu
[3] Tim Taylor Department of Chemical Engineering, Kansas State University, Manhattan, KS 66506, USA; jiahanli@ksu.edu (J.L.); edgarjh@ksu.edu (J.H.E.)
[4] Department of Electrical and Computer Engineering, Auburn University, Auburn, AL 36849, USA; taba.adib@auburn.edu (A.T.); mzm0185@auburn.edu (M.M.-S.)
* Correspondence: bzn0023@auburn.edu (B.-I.N.); sdai@auburn.edu (S.D.)

Supplementary information S1 - Optical constants of hBN

The infrared optical properties of hBN have been thoroughly explored through experimental assessments and first-principal calculations based on theoretical computations. This is a wide consensus that the permittivity in both in-plane and out-of-plane directions can each be described by Lorentz model as below [1]:

$$\varepsilon_j(\omega) = \varepsilon_{\infty,j} + \varepsilon_{\infty,j}\frac{(\omega_{LO,j})^2-(\omega_{TO,j})^2}{(\omega_{TO,j})^2-\omega^2-i\omega\Gamma_j} \quad \text{(S1)}$$

Where $\varepsilon_j(\omega)$ indicates the in-plane (j=x=y) and out-of-plane (j=z) dielectric functions of perpendicular and parallel directions along the optical z-axis, respectively. $\varepsilon_{\infty,j}$ means the limiting high-frequency permittivity. The $\omega_{LO,j}$ and $\omega_{TO,j}$ are longitudinal optical (LO) phonon frequency, and transverse optical (TO) phonon frequency, respectively. Γ_j is optical phonon broadening (damping constant). These parameters are experimentally and theoretically obtained by many researchers [2,3]: $\varepsilon_{\infty,x}$ = 4.9, $\omega_{LO,x}$ = 1614 cm^{-1}, $\omega_{TO,x}$ = 1360 cm^{-1}, Γ_x = 7 cm^{-1}, $\varepsilon_{\infty,z}$ = 2.95, $\omega_{LO,z}$ = 825 cm^{-1}, $\omega_{TO,z}$ = 760 cm^{-1}, and Γ_z = 3 cm^{-1}. The calculated hBN permittivity has been plotted in Figure S1.

Table S1 Parameters for the calculated complex dielectric function of hBN.

i	$\varepsilon_{\infty,i}$	$\omega_{LO,i}$ (cm^{-1})	$\omega_{TO,i}$ (cm^{-1})	Γ_i (cm^{-1})
x (or y)	4.9	1614	1360	7
z	2.95	825	760	3

Supplementary information S2 - Infrared reflectivity of hBN/ZrS_2/hBN heterostructures

The HPhP dispersion can be modeled by plotting the imaginary part of complex reflectivity Im r_p (k, ω) of the air/hBN/SiO_2/Si, air/top hBN/10 nm ZrS_2/bottom hBN/SiO_2/Si, and air/top hBN/43 nm ZrS_2/bottom hBN/SiO_2/Si, where the complex momentum of HPhPs is k and frequency is ω." At that time, the most electric field of HPhPs is confined to the interfacial layers of hBN. Thus, the Im r_p (k, ω) of the multilayer case can be calculated through Fresnel equation as shown in Figure 3 (main text) [2,4,5]:

$$r_p = \frac{r_{12}+r_{23}e^{i2k_2^z d}}{1+r_{12}r_{23}e^{i2k_2^z d}} \tag{S2}$$

$$r_{12} = \frac{\varepsilon_x k_1^z - \varepsilon_1 k_2^z}{\varepsilon_x k_1^z + \varepsilon_1 k_2^z} \tag{S3}$$

$$r_{23} = \frac{\varepsilon_3 k_2^z - \varepsilon_x k_3^z}{\varepsilon_3 k_2^z + \varepsilon_x k_3^z} \quad , \tag{S4}$$

where the subscripts "1", "2", and "3" indicate the layer of air, hBN, and SiO_2, respectively. Eq. S3 and S4 indicated the reflectivity of the air/hBN and hBN/SiO_2 interface, respectively. d is the thickness of the hBN and k^z represents the out-of-plane momentum in each layer i: $k_i^z = \sqrt{\varepsilon_i \frac{\omega^2}{c^2} - k^2}$, i = Air and SiO_2. For the uniaxial anisotropy of hBN, $k_{hBN}^z = \sqrt{\varepsilon_x \frac{\omega^2}{c^2} - \frac{\varepsilon_x}{\varepsilon_z} k^2}$.

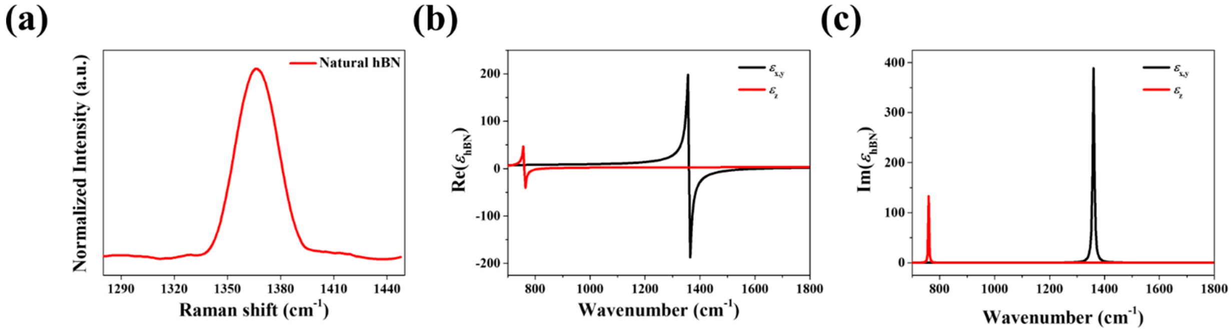

Figure S1 | Optical information of hBN. (a) Raman spectrum of the natural hBN. **(b), (c)** Real and imaginary parts of the dielectric function for natural hBN.

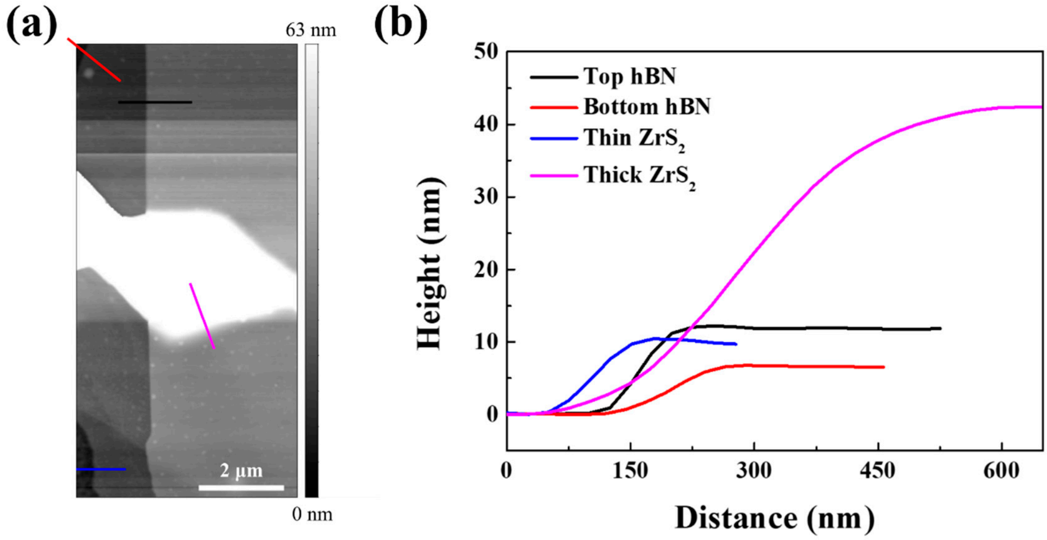

Figure S2 | Morphology characterization. (a) AFM image of vdW heterostructures in this work. **(b)** Height profiles were obtained from several dashed lines in the left panel (a).

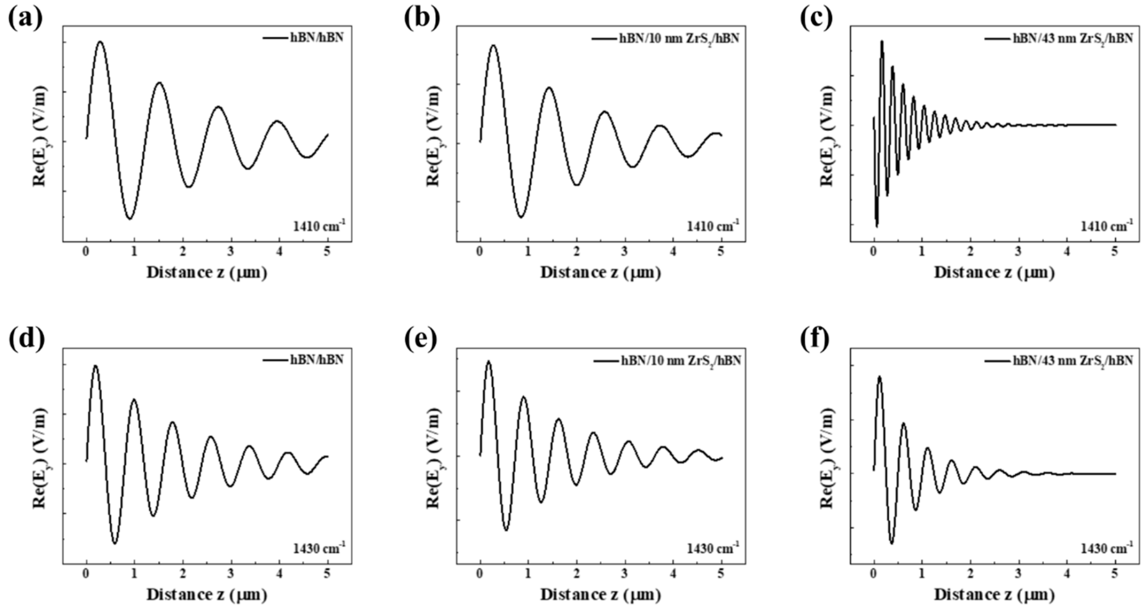

Figure S3 | Line profiles of Re(E_z) from COMSOL simulation. hBN/hBN, hBN/10 nm ZrS_2/hBN, and hBN/43 nm ZrS_2/hBN at ω = 1410 (a, b, c) and 1430 cm^{-1} (d, e, f).

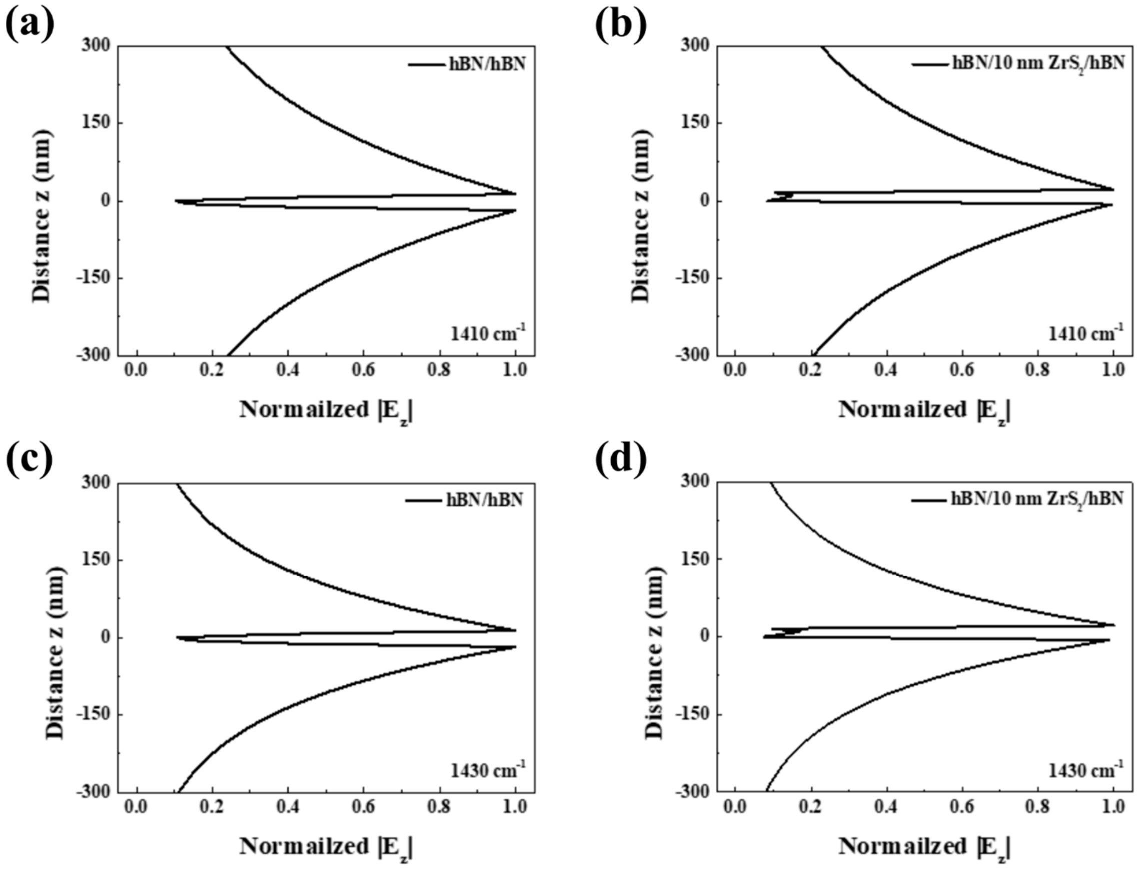

Figure S4 | Theoretical EM field distribution $|E_z|$ profiles. hBN/hBN and hBN/10 nm ZrS_2/hBN at ω = 1410 cm^{-1} (a, b) and 1430 cm^{-1} (c, d).

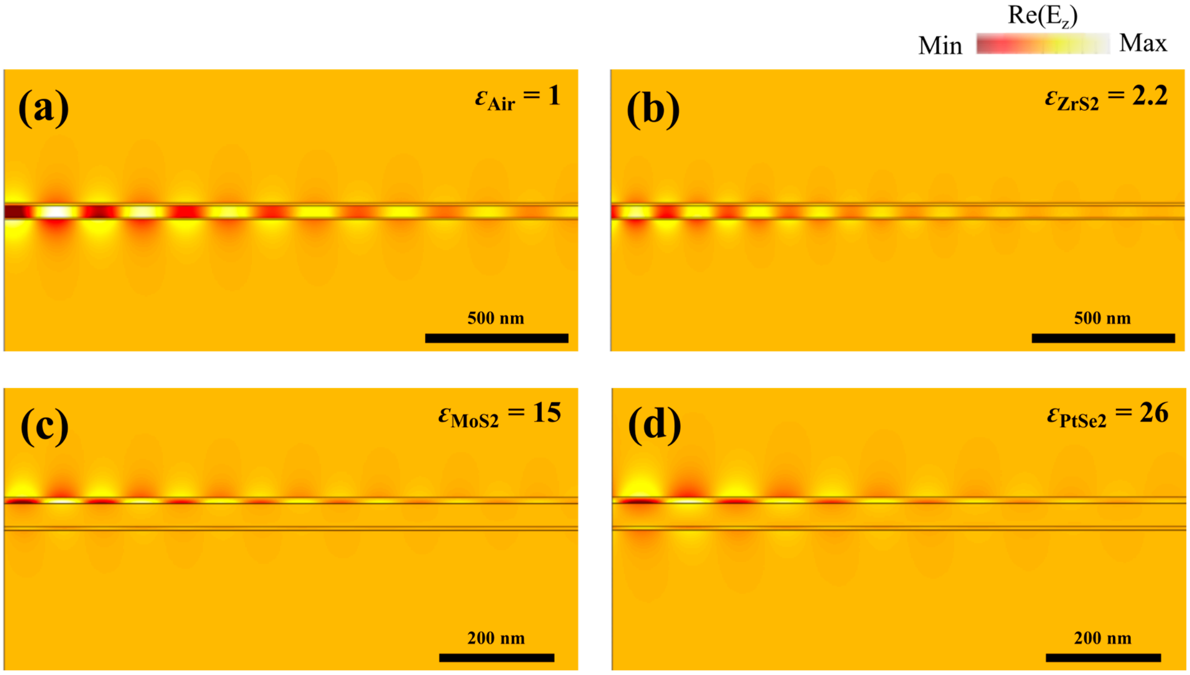

Figure S5 | Permittivity dependence. Full-wave EM numerical simulation of vdW heterostructures inserting different middle layers: 43 nm of (a) Air, (b) ZrS_2, (c) MoS_2, and (d) $PtSe_2$ at the frequency ω = 1410 cm^{-1}.

Reference

1. Caldwell, J.D.; Kretinin, A.V.; Chen, Y.G.; Giannini, V.; Fogler, M.M.; Francescato, Y.; Ellis, C.T.; Tischler, J.G.; Woods, C.R.; Giles, A.J.; et al. Sub-diffractional volume-confined polaritons in the natural hyperbolic material hexagonal boron nitride. *Nature Communications* **2014**, *5*, 5221, doi:10.1038/ncomms6221.
2. Dai, S.; Fei, Z.; Ma, Q.; Rodin, A.S.; Wagner, M.; McLeod, A.S.; Liu, M.K.; Gannett, W.; Regan, W.; Watanabe, K.; et al. Tunable Phonon Polaritons in Atomically Thin van der Waals Crystals of Boron Nitride. *Science* **2014**, *343*, 1125-1129, doi:10.1126/science.1246833.
3. Giles, A.J.; Dai, S.Y.; Vurgaftman, I.; Man, T.H.; Liu, S.; Lindsay, L.; Ellis, C.T.; Assefa, N.; Chatzakis, I.; Reinecke, T.L.; et al. Ultralow-loss polaritons in isotopically pure boron nitride. *Nature Materials* **2018**, *17*, 134-+, doi:10.1038/nmat5047.
4. Dai, S.Y.; Tymchenko, M.; Xu, Z.Q.; Tran, T.T.; Yang, Y.F.; Ma, Q.; Watanabe, K.; Taniguchi, T.; Jarillo-Herrero, P.; Aharonovich, I.; et al. Nanostructure Diagnosis with Hyperbolic Phonon Polaritons in Hexagonal Boron Nitride. *Nano Letters* **2018**, *18*, 5205-5210, doi:10.1021/acs.nanolett.8b02162.
5. Chen, M.; Zhong, Y.; Harris, E.; Li, J.; Zheng, Z.; Chen, H.; Wu, J.S.; Jarillo-Herrero, P.; Ma, Q.; Edgar, J.H.; et al. Van der Waals isotope heterostructures for engineering phonon polariton dispersions. *Nature Communications* **2023**, *14*, 4782, doi:10.1038/s41467-023-40449-w.
